# Placental Galectin-2 Expression in Gestational Diabetes: A Systematic, Histological Analysis

**DOI:** 10.3390/ijms21072404

**Published:** 2020-03-31

**Authors:** Paula Hepp, Laura Unverdorben, Stefan Hutter, Christina Kuhn, Nina Ditsch, Eva Groß, Sven Mahner, Udo Jeschke, Julia Knabl, Helene H. Heidegger

**Affiliations:** 1 Department of Gynecology and Obstetrics, University Hospital, LMU Munich, Maistraße 11, 80337 Munich, Germanyl.unverdorben@krk-aoe.de (L.U.); hutter.stefan@googlemail.com (S.H.); christina.kuhn@med.uni-muenchen.de (C.K.); nina.ditsch@uk-augsburg.de (N.D.); eva.gross@med.uni-muenchen.de (E.G.); sven.mahner@med.uni-muenchen.de (S.M.); julia.knabl@gmx.de (J.K.); Helene.heidegger@med.uni-muenchen.de (H.H.H.); 2 Department of Obstetrics and Gynecology, Klinik Altötting, 84503 Altötting, Germany; 3 Department of Obstetrics and Gynecology, University Hospital Augsburg, 86156 Augsburg, Germany; 4 Department of Obstetrics, Klinik Hallerwiese, 90419 Nürnberg, Germany

**Keywords:** galectin-2, gestational diabetes (GDM), placenta, insulin resistance, chronic low-grade inflammation, metabolism

## Abstract

Gestational diabetes mellitus (GDM) is the most common pregnancy-associated metabolic disorder that negatively impacts on the health of both mothers and their offspring in the long-term. The molecular mechanisms involved are not fully understood. As in other states of insulin resistance, a disproportionate immune response in GDM leads to a state of chronic low-grade inflammation. Galectin-2 exerts regulatory effects on different immune cells. This study investigated galectin-2 expression in the placenta of 40 GDM patients and 40 controls, in a sex-specific manner. Immunohistochemistry was used for semi-quantitative analysis of expression strength. The phenotypes of galectin-2 expressing cells were characterized through double immunofluorescence. We found a significant up-regulation of galectin-2 in the fetal syncytiotrophoblast, as well as in the maternal decidua of GDM placentas. Double staining showed a strong galectin-2 expression in extra villous trophoblast cells and fetal endothelial cells in GDM. These findings present the first systematic investigation of galectin-2 in GDM. The findings contribute to the emerging understanding of the role of immunomodulation and inflammation in GDM and of galectin-2 itself. This might also have implications for the long-term cardiovascular health of the offspring.

## 1. Introduction

Galectin proteins are those members of the large lectin family, which by definition contain a specific amino-acid sequence—the so-called carbohydrate recognition domain (CRD)—and thus bind the β-galactoside sugar domains [1,2]. The ever-growing protein family is currently classified according to their molecular structure, more specifically the number of different CRDs—prototype galectins contain only one CRD and, therefore, tend to form dimers in order to fulfil cross-linking functions. Tandem repeat galectins contain two different CRDs, while the chimera-type galectin contain one CRD and one N-terminal non-CRD domain within one molecule [3].

Extensive research into the physiological significance and functioning of galectins has been conducted over the past decades. The binding of specific Gal(β1-4)GlcNAc or Gal(β1-3)GlcNAc-terminating oligosaccharides in the CRD and the following crosslinking, form one basis of galectin action [4,5,6]. One specific galectin fulfils numerous functions that might not only be different but even contrary, depending on tissue type and intra- or extracellular location [1,7]. By now 16 different galectins have been identified in humans and the majority are expressed in placental tissue [8]. Other organ systems that show a strong galectin involvement include the intestine and immune cells [9,10]. Galectins are modulators of ubiquitous physiological pathways, such as cell migration, cell-to-cell communication, apoptosis, and proliferation [11,12]. Therefore, they play an important role in numerous more complex processes, most prominently angiogenesis, metabolism, and immunomodulation [10,13,14]. These in turn are of immense importance for pathologies, including cancer progression, cardiovascular disease, and of course innumerable inflammatory disorders—all of which had galectin dysregulations that have been linked with [15,16,17,18].

Angiogenesis and immunomodulation form the basis of many mechanisms in physiological pregnancy, including implantation, placentation, and feto-maternal tolerance. It is, therefore, not surprising that galectins have become a focus in the field of reproductive biology. Galectins-1 and -3 are best studied in this context. Using extensive mouse models Blois et al. [19] were able to show the pivotal role of galectin-1 in feto-maternal tolerance. Knockout resulted in higher rates of fetal loss, which could to a certain extend be reversed through treatment with recombinant galectin-1. The pathway through which galectin-1 acts are brought forward here includes the induction of tolerogenic dendritic cells and regulatory T-cells. These results were underpinned by further studies in humans [20]. A galectin-3 knock-out similarly hindered implantation in mouse models [21].

The pathophysiological connection between faulty implantation, inadequate immune response, and numerous pregnancy disorders is well-known and various galectin dysregulation are found in pathologies, including preeclampsia (PE), intrauterine growth restriction (IUGR), and HELLP-syndrome [22,23,24]. In PE, molecular research has come as far as identifying galectin-13 (also known as placental protein-13 (PP-13)) as a clinically relevant predictive marker for the development of PE [25].

Another pregnancy disorder showing galectin involvement is gestational diabetes mellitus (GDM). It is the most common metabolic-disorder in pregnancy and—contrary to most obstetric morbidities—its prevalence has steadily increased over the past decades [26,27,28]. It has grave implications in terms of perinatal complications, due to the associated risk of PE, fetal macrosomia, and C-sections [29,30]. The burden of GDM is further aggravated by the long-term morbidity it causes—mother and offspring stand a significantly higher risk of developing type 2 diabetes (DM2), adiposity, metabolic syndrome, and cardiovascular disease [31,32,33,34,35].

Similar to other pregnancy disorders, the pathophysiology of gestational diabetes involves the dysregulation of immunoregulation. GDM manifests when the anti-insulinemic effect of placental steroid hormones is not balanced with an increased maternal insulin production in beta-cells [36]. Furthermore, it was found that GDM resembles a state of insulin resistance similar to DM2 [37]. This is also accompanied by a chronic state of low-grade inflammation [38]; for example, interleukin (IL) 6 and tissue inhibitor of metalloprotease 1 (TIMP-1), a marker for low-grade inflammation, were shown to be elevated in GDM [39,40]. Remarkably, epidemiological studies were able to show a significant association between these markers and cardiovascular risk in the general population and a GDM cohort, respectively [41,42].

As modulators of inflammation, some members of the galectin family have previously been described in GDM. Results of these studies showed that galectin-1, -3, and -13 are significantly dysregulated in the placentas of GDM patients, and galectin-13 has recently been tested as a potential biomarker for GDM-screening [43,44,45,46].

Galectin-2, on the other hand, is generally not as well understood. It belongs to the prototype galectins, thus, contains only one CRD and typically forms a homo-dimer [2,47]. Like other members of the galectin family, it is involved in immunomodulation. Interestingly, it has been linked with pro- as well as anti-inflammatory actions [48,49]. Previous studies on placental tissue showed a significant decrease of galectin-2 in miscarriages, preeclampsia, and male IUGR cases [23,50,51].

To the best of our knowledge so far there are no reports on the role of galectin-2 in GDM and only few reports concerning other forms of diabetes are available. One large genetic–epidemiological study of 3,272 British women was conducted by Christensen et al. [52]. It showed that the functional A:T rs7291467 single nucleoid polymorphism (SNP), which in the galectin-2 gene (LGALS2) affects the gene’s transcription level [53], is associated with higher fasting levels of insulin and glucose. Interestingly, the same SNP was found to be associated with a risk of cardiovascular disease in different Asian population [54,55], but no association was found in European population studies [56,57].

Considering what is currently known as GDM’s pathophysiology and functions of galectins, we hypothesized, that galectin-2 expression is altered in GDM placentas.

This study presents a sex-specific, systematic analysis of galectin-2 expression in control and GDM placentas, as well as a characterization of galectin-2 expressing phenotypes. The aim of which is to establish whether galectin-2 dysregulation plays a part in the pathophysiology of GDM. This might be a starting point for further investigation into the role of galectin-2 in metabolic dysregulation.

We found galectin-2 to be significantly upregulated in both fetal syncytiotrophoblast (SCT) and maternal decidua of GDM placentas. The sex-specific analysis revealed no significant differences between female and male placentas.

## 2. Results

A systematic and sex-specific immunohistochemical and immunofluorescent analysis of galectin-2 expression was conducted on 40 healthy (20 female, 20 male foetuses) and 40 GDM (20 female, 20 male foetuses) placentas.

### 2.1. There Are No Sex-Specific Differences in Galectin-2 Expression

**Figure 1:**
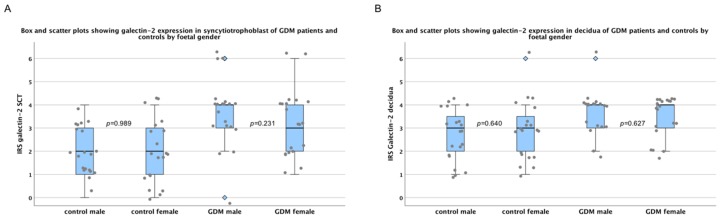
Sex-disaggregated galectin-2 expression. Box and scatter plots show the immunoreactivity score (IRS) for galectin-2 expression by fetal gender in syncytiotrophoblast (SCT; **A**) and decidua (**B**). The range between the 25th and 75th percentiles is represented by the boxes, with the horizontal line showing median. The bars indicate the 5th and 95th percentiles. Blue diamonds indicate values more than 1.5-times the boxes’ lengths. Grey dots represent singular data points. There was no statistically significant difference between the male and female groups, as shown by the *p*-values.

Since sex-specific differences are common in placental disorders, sex-disaggregated data collection was used throughout the study. Furthermore, statistical analysis was applied to check for sex-specific differences in galectin-2 expression, within the control group as well as the GDM group. However, no significant difference was found between male and female foetuses concerning the galectin-2 expression in either group. As can be seen in Figure 1, this was the case in fetal syncytiotrophoblast, as well as maternal decidual tissue.

### 2.2. Galectin-2 Expression Is Upregulated in the Fetal Syncytiotrophoblast of GDM Placentas

**Figure 2:**
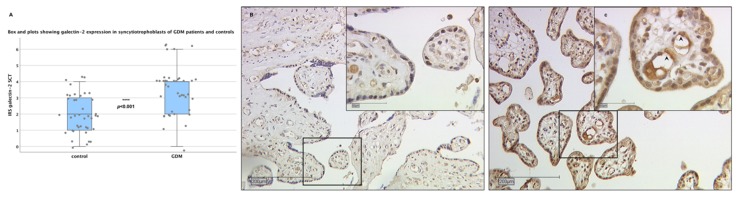
Galectin-2 expression in syncytiotrophoblast (SCT) of control and gestational diabetes mellitus (GDM) placentas. Box and scatter plots (**A**) showing the IRS for galectin-2 expression in SCT to be significantly higher in GDM placentas (*p* < 0.001). See Figure 1 for a detailed plot explanation. Pictures show representative slides for immunohistochemical staining of galectin-2 in the SCT of the control (**B**) and GDM (**C**) placentas. The scale bar equals 200 μm in full size images and 50µm in inserts. Black rectangles indicate the section seen at higher magnification (minuscular letters). Arrow heads show strongly stained fetal endothelial cells.

Based on the first statistical analysis of sex-specific differences described above, we pooled female and male controls and GDM placentas for the following analyses. In the fetal syncytiotrophoblasts, the immunohistochemical evaluation detected a low (IRS: 2) expression of galectin-2 in control placentas. As can be seen in Figure 2, this expression was significantly upregulated in the SCT of GDM placentas (*p* < 0.001), with the median IRS coming to 4.

### 2.3. Galectin-2 Expression Is Upregulated in the Maternal Decidua of GDM Placentas

**Figure 3:**
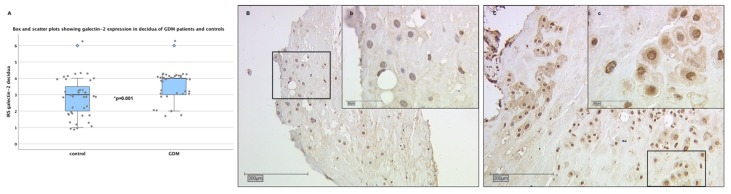
Galectin-2 expression in the maternal decidua of control and GDM placentas. Box and scatter plots (**A**) showing the IRS for galectin-2 expression in decidua to be significantly higher in GDM placentas (*p* = 0.001). See Figure 1 for a detailed box plot explanation. Pictures show representative slides for immunohistochemical staining of galectin-2, in the maternal decidua of the control (**B**) and the GDM (**C**) placentas. The scale bar equals 200 μm in full size images and 50µm in inserts. Black rectangles indicate the section seen at a higher magnification (minuscular letters).

A similar pattern was found in the maternal decidua. The galectin-2 expression in the control group was low to moderate, with a median IRS of 3. Like the fetal syncytiotrophoblast, the maternal decidual tissue of the GDM group showed a significantly higher galectin-2 expression than the controls (*p* = 0.01; median IRS: 4; see Figure 3).

### 2.4. There are No Significant Differences in Galectin-2 Expression between Normal and Overweight Pregnancies

**Figure 4:**
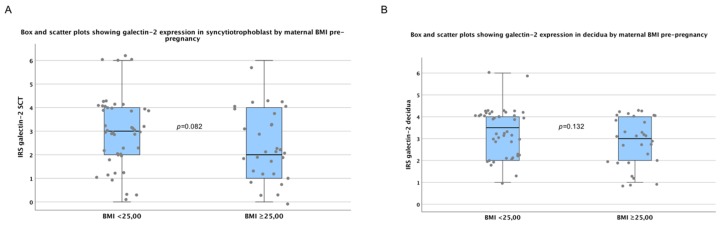
Galectin-2 expression by maternal body mass index (BMI) pre-pregnancy. There was no significant difference between the groups. For detailed plot explanation see Figure 1.

Due to the fact that maternal body mass index (BMI), prior to pregnancies was significantly higher in the GDM group compared to controls, we conducted statistical analysis to investigate the influence of BMI on placental galecetin-2 expression. Our testing showed no significant difference between galectin-2 expression in subjects with low and normal BMI (<25kg/m^2^) and in overweight and obese (BMI ≥25kg/m^2^) patients. This was the case in fetal SCT (*p* = 0.082), as well maternal decidua (*p* = 0.132) tissue (Figure 4). Therefore, BMI did not seem to be a confounder of the differences in placental galectin-2 expression found in this study.

### 2.5. Identification of Galectin-2 Expressing Cells by Immunofluorescence Double Staining

**Figure 5:**
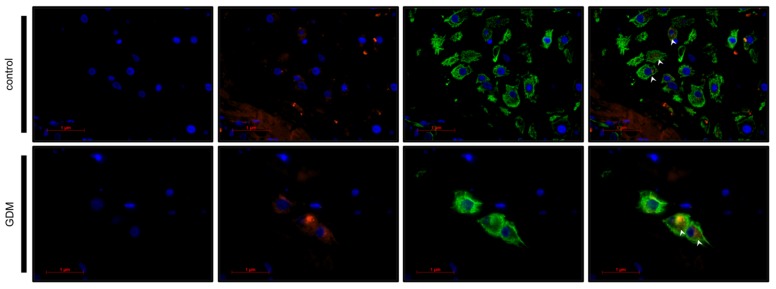
Double immunofluorescence phenotyping of decidual cell. Nuclei are stained blue using DAPI. Galectin-2, bound by Cy-3-labled secondary antibody, is stained red. CK7, bound by Cy-2-labeled secondary antibody is stained green, marking the extra-villous trophoblast (EVT) cells. Arrows heads indicate the merging expression of CK7 and galectin-2 visible as yellow. The scale bar equals 1 μm.

**Figure 6:**
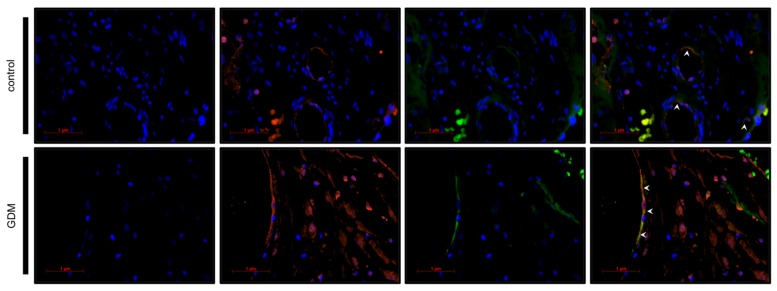
Double immunofluorescence phenotyping of villus cells. Nuclei are stained blue using DAPI. Galectin-2, bound by Cy-3-labled secondary antibody, is stained red. CD31, bound by Cy-2-labeled secondary antibody is stained green, marking fetal endothelial cells. Arrows heads indicate the merging expression of CD31 and galectin-2, visible as yellow. The scale bar equals 1 μm.

In order to identify the phenotype of galectin-2 expressing cells, immunofluorescence double staining followed by fluorescent microscopy were carried out (see Figure 5 and Figure 6; for magnified sections, see Figure S1). Cytokeratin 7 (CK7) and cluster of differentiation (CD) 31 were used as markers for the extra-villous trophoblast cells (EVTs) and fetal endothelial cells, respectively [58,59].

Microscopic evaluation confirmed CK7 and galectin-2 expression in the same cell, thereby, identifying fetal EVTs as the predominant galectin-2 expressing cell type in the maternal decidua of the GDM, as well as the control group (see Table 1). The galectin-2 expression in GDM placentas appeared to be more intense, compared to the control.

Similarly, fluorescence microscopy demonstrated the co-expression of CD31 and galectin-2, thus, verifying galectin-2 expression by endothelial cells. Again galectin-2 expression appeared to be more intense in GDM. There were also numerous CD31 negative cells in the villus mesoderm of GDM cases that expressed galectin-2.

## 3. Discussion

Galectins are major players in the regulation of immunomodulation and metabolism and their dysregulation takes up an important role in numerous pathologies, including GDM. Our study corroborates the current evidence by showing an increased expression of galectin-2 in the syncytiotrophoblast, EVT, and the fetal endothelial cells of GDM patients. This was the first investigation to establish a role of galectin-2 dysregulation in the pathophysiology of GDM.

Blois et al. [44] previously showed a dysregulation of galectin-1 in GDM patients. While peripheral galectin-1 serum levels in GDM patients were lower compared to controls, expression in the placenta was significantly increased. Since galectin-1 is generally considered to be anti-inflammatory, it was hypothesized that its upregulation might be a reaction to the state of chronic inflammation present in GDM, aiming to restore balance [10,11,44].

Both galectin-1 and galectin-2 are prototype galectins and showed a 47% structural overlap in humans. However, in terms of T-cell interaction, the two galectins bind distinct glycoproteins and are, hence, thought to induce different cellular pathways [60]. Therefore, it should not be assumed that their involvement in immunoregulation could be seen as one. Moreover, a large number of studies have been conducted on galectin-1′s receptor binding and transcription modulation in trophoblast cells, while this kind of information is still largely lacking for galectin-2 [8].

Nevertheless, information on the galectin-2 function is accumulating through investigations in models of auto-immune diseases. In a model of auto-immune dermatitis, Loser et al. [61] showed a comparable upregulation of galectin-2 in the affected skin tissue. In the same study treatment, galectin-2 reduced the number of activated CD8+ T-cells while the regulatory T-cells (CD4+) were not affected [61]. Similarly, galectin-2 shifted T-cell cytokine profiles towards a T-helper-cell (Th) 2 phenotype downregulation of interferon (INF) γ, tumor necrosis factor (TNF) α, and upregulation of IL-5 [60]. Based on these findings, further investigations into the therapeutic potential of galectin-2 were conducted; these showed a significant reduction of inflammation in acute and chronic mice colitis-models [48]. In activated neutrophils, galectin-2 induced externalization of phosphatidylserine, leading to phagocytosis [62]. In light of these anti-inflammatory qualities, it seemed reasonable to consider the increased placental expression of galectin-2 as a reaction to the state of chronic inflammation present in GDM.

Very recently, Maeda et al. [63] were the first to show that galectin-2 involvement is direct in adequate insulin secretion of pancreatic beta-cells. In their study, disruption of galectin-2 resulted in reduced insulin secretion. Should galectin-2 also be upregulated systemically in GDM cases, it might be hypothesized that this upregulation aims at stabilizing insulin secretion in beta-cells. Therefore, further investigations into galectin-2 on protein and DNA level in mothers as well as offspring are necessary.

On the other hand, Yildirim et al. [49] recently showed pro-inflammatory effects of galectin-2 via polarization of M0 macrophages into a proinflammatory M1 state, which is accompanied by increased concentrations of inflammatory cytokines, including IL-6 and TNFa. This clarified that immunomodulation through galectins is extremely complex and the mechanisms induced even by one member of the galectin family vary, depending on the cell type [64].

In normal pregnancy, macrophages account for around 20% of decidual leukocytes. Typically, a shift from M1 to M2 phenotype can be observed on a cellular as well as epigenetic level [65]. Therefore, a second hypothesis emerges. The increased galectin-2 expression in GDM characterized in this study might contribute to the proinflammatory milieu of insulin resistance via macrophage dysregulation. Further studies on macrophage phenotype and cytokine profiles in GDM should be conducted to gain further insights into the complexity of galectin-2 function in this context.

Nonetheless, this complexity seems to somewhat mirror the complexity of alterations in cytokine profile in GDM mothers recently characterized by Ategbo et al. [66]. Both an increase of proinflammatory IL-6 and TNFα and the downregulation of Th1/Th2 ratio described in their paper, could hypothetically be mediated (at least partially) by an increased galectin-2 expression. It must be noted, however, that the cytokine and T-cell profile in macrocosmic offspring differed greatly—a fact that yet needs to be clarified [66].

Through double immunofluorescence we were able to show that the upregulation of galectin-2 is not restricted to the trophoblast, but directly affects fetal endothelial cells, as well. Thus, implications for angiogenesis and cardiovascular disease should also be considered.

An induction of the M1 phenotype (as described above) goes hand in hand with reduced numbers of M2 macrophages, which are important producers of pro-arteriogenic factors like vascular endothelial growth factor (VEGF) A or matrix metalloproteinase (MMP) 2 [49]. This might be a causal link through which galectin-2 exerts its anti-arteriogenic effect, previously described in patients with coronary heart disease (CHD) and CHD mouse models [67,68]. More broadly, cardiovascular diseases are generally linked to inflammation and increased levels of IL-6 were shown to be significantly associated with a risk of CHD [41].

Taking into consideration the strong expression of galectin-2 in fetal endothelial cells and surrounding CD31-negative cells of GDM placentas (see Figure 5), this might well be relevant for the development of life-long cardiovascular risk of the offspring. Hypothetically, an increased concentration of galectin-2 could lead to a perivascular M1 phenotype induction, which in turn could lead to a pro-inflammatory state that interferes with adequate vascularization.

In conclusion, the present study establishes the role of galectin-2 dysregulation in the pathophysiology of GDM. Due to the descriptive nature of our findings the question arises whether the increased galectin-2 expression is a reaction to the inflammatory state of GDM or whether it might contribute to its development. Considering current knowledge on galectin-2 function, either conclusion seems somewhat reasonable. Hence, further research is urgently needed to clarify its role in GDM and potentially reveal its therapeutic implications.

## 4. Materials and Methods

### 4.1. Tissue Samples

After the study design was approved by the LMU ethics committee, 40 GDM patients and 40 healthy expectant mothers (control) were chosen to participate. Fetal gender was balanced in both groups. Written consent was obtained from all participants in advance. To be included in the study, all participants underwent an oral glucose tolerance test (oGTT) between week 24 and 28 of their pregnancy. The diagnosis of GDM was based on the criteria of the German society for Diabetes Mellitus (two measurements above limits—fasting glucose >90 mg/dL, 1 h > 180 mg/dL, and 2 h > 155 mg/dL) [69]. Clinical and epidemiological data of the study cohort is depicted in Table 2 and Table 3.

Tissue samples (2 × 2 × 2 cm^3^) from a central cotyledon of the participants’ placentas were obtained directly after birth. The areas of sampling contained maternal decidua, fetal syncytiotrophoblast, and amniotic epithelia. Macroscopically they were sufficiently supplied with blood, while areas with signs of calcification, bleeding, or ischemia were avoided. After 24 h of fixation in 4% buffered formalin solution, the tissue samples were embedded in paraffin for long-term storage.

### 4.2. Immunohistochemistry

#### 4.2.1. Staining

**Figure 7:**
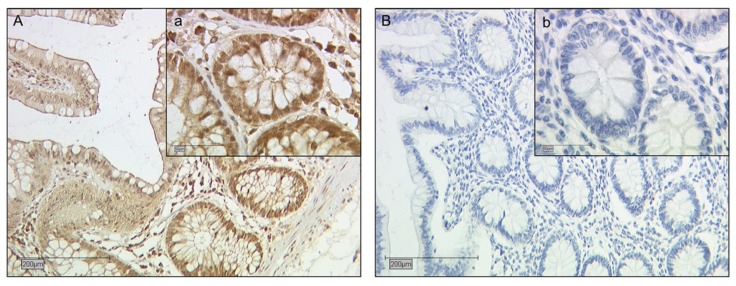
Immunohistochemistry control staining. Colon tissue was used as positive (**A**, **a**) as well as negative (**B**, **b**) control, for anti-galectin-2-antibody.

The immunohistochemical staining was based on a detailed protocol recently published by Hutter et al. [71]. A general overview is given in the following. After removal of paraffin in a Roticlear (Carl Roth, Karlsruhe, Germany) bath, endogenous peroxidase activity was blocked using 3% H_2_O_2_. Second, the protein epitopes were demasked by high-pressure sodium citrate (pH 6.0) treatment. Blocking solution (ZytoChem Plus HRP Polymer System, Zytomed Systems GmBH, Berlin, Germany) was applied to prevent unspecific antigen–antibody interaction. Thereafter, the slides were incubated with primary antibodies—anti-galectin-2-antibody (polyclonal rabbit IgG, concentration 0.05mg/mL, NBP1-89690, Novus Biologicals, Minneapolis, USA) dissolved in PBS at 1:200 dilution for 16 h, at 4 °C. After washing the slides with PBS they were treated with Post Block (Reagent 2, ZytoChem Plus HRP Polymer System mouse/rabbit, Zytomed) for 20 min, followed by HRP Polymer (Reagent 3, ZytoChem Plus HRP Polymer System mouse/rabbit, Zytomed) for 30 min. Visualization was achieved by applying chromogen 3,3′–diaminoenzidine (DAB; Dako, Glostrup, Denmark). Positive and negative control staining was carried out on human colon tissue, alongside each round of staining (see Figure 7). Positive controls served to ensure viability of the antibody. Negative controls were used to rule out any unspecific staining. In order to achieve this negative control, serum (negative control for super sensitive rabbit antibodies, rabbit IgG, Biogenics, Fremont, USA) containing anti-rabbit-Igs was applied instead of the primary antibody. Mayer’s hemalum was used for counterstaining. After dehydration in an ascending series of alcohol and Roticlear (Carl Roth, Karlsruhe, Germany) treatment, the slides were cover slipped.

#### 4.2.2. Evaluation

All samples were evaluated under a Leitz Diaplan microscope using 10-fold and 40-fold objectives (see also corresponding photographs in result section). The semi-quantitative Immunoreactivity Score (IRS) [72] was used to evaluate tissue staining—cell staining intensity (0: none; 1: weak; 2: moderate; 3: strong) and the percentage of positively stained cells (0: no staining; 1: <10% of the cells; 2: 11–50%; 3: 51–80%; 4: >80) were evaluated separately and, thereafter, the values were multiplied, resulting in an IRS between 0 and 12 for each slide. One slide of high staining quality was evaluated per participant on which 3–5 microscopic fields and a minimum of 100 cells were counted. All slides were evaluated by two independent observers, with no more than two sessions per observer. If the observers came to diverging conclusions, the sample was re-evaluated and discussed until reaching one conclusive result.

### 4.3. Double Immunofluorescence

For the phenotypical characterization of galectin-2 expressing cells double immunofluorescence was conducted with CK7 as a marker for extra villous trophoblast cells [58] and CD31 as a marker for fetal endothelial cells [59].

After removal of paraffin and de-masking of all protein epitopes (see Immunohistochemistry), blocking solution (Ultra V–Block, Thermo Scientific, Lab Vision, Fremont, CA, USA) was applied for 15 min, in order to prevent unspecific antigen–antibody interaction. The slides were then incubated with the primary antibody mixtures (see Table 4 for details). Subsequently the fluorescent secondary antibodies were applied for 30 min (see Table 4 for details). Following the antibody incubation, the slides were cover slipped using mounting buffer (Vector Laboratories, Burlingame USA), which contains DAPI for nuclear counterstaining. Light exposure was kept to a minimum during the covering process. The fluorescent Axioskop photomicroscope (Zeiss, Oberkochen, Germany) was used for evaluation of fluorescent staining. Photographs were taken with a digital Axiocam camera system (Zeiss, Oberkochen, Germany). Evaluation and documentation were done under a 63-fold objective. For quantification of the relative share of EVT cells of the galectin-2 expression, the number of galectin-2 expressing cells, and that of CK7-galectin-2 double positive cells were counted on the generated pictures.

### 4.4. Statistical Analysis

IBM SPPS Statistics (Version 22.0. for Windows, Armonk, NY, USA) was used for data collection, analysis, and visualization. The non-parametric Man-Whitney-U test was used for the analysis of statistical significance, which was assumed to be at *p* < 0.05.

## Figures and Tables

**Table 1:** Quantification of galectin-2 expressing cells.

	Number of Galectin-2 Positive Cells Evaluated	Galectin-2 and CK7 Double Positive Cells	Percentage of Galectin-2 and CK7 Positive Cells
Control	41	39	95,1 %
GDM	79	77	97,5 %

**Table 2:** Clinical and epidemiological data of study cohort by fetal gender. The data were analysed using the Kruskal Wallis Test.

	GDM		Control		*p*-Value
	Male	Female	Male	Female	
Birthweight (g)	3662.1 ± 562	3635.9 ± 661	3339.8 ± 568	3294 ± 440	*p* = 0.019 *
Duration of gestation at delivery (weeks)	39.67 ± 1.30	39.83 ± 1.40	39.80 ± 1.54	39.75 ± 1.16	*p* = 0.943
Maternal BMI pre-pregnancy (kg/m^2^)	29.38 ± 8.03	26.96 ± 4.73	21.92 ± 3.97	25.04 ± 7.90	*p* < 0.001 *
pH in umbilical artery	7.30 ± 0.07	7.30 ± 0.10	7.28 ± 0.10	7.30 ± 0.08	*p* = 0.826
Maternal Age (years)	31.46 ± 4.12	33.21 ± 5.33	30.30 ± 6.11	32.00 ± 6.13	*p* = 0.177

Statistically significant differences are marked with an asterisk (*).

**Table 3:** Number of GDM and control subjects per BMI group [70].

	Number of Patients in GDM Group	Number of Patients in Control Group
Underweight (BMI < 18.5 kg/m^2^)	0	4
Normal BMI (18.5–24.9 kg/m^2^)	16	25
Overweight (25.0–29.9 kg/m^2^)	10	3
Obese (≥30.0 kg/m^2^)	12	5

**Table 4:** Antibody features used for double-immunofluorescence.

Antibody	Dilution	Incubation	Manufacturer
Galectin-2—polyclonal Rabbit IgG	1:200	16 h at 4 °C	Novus Biologicals—NBP1-89690
CK7—Clone OVTL Mouse IgG	1:30	16 h at 4 °C	Novocastra—NCL-L-CK7-OVTL
CD31—Clone JC/70A Mouse IgG	1:50	16 h at 4 °C	Abcam—ab9498
Cy-2-labelled goat-anti-rabbit	1:100	30 min at RT	Dianova—115-226-062
Cy-3-labelled goat-anti-mouse	1:500	30 min at RT	Dianova—111-165-144

## Supplementary Materials

Supplementary materials can be found at https://www.mdpi.com/1422-0067/21/7/2404/s1.

## Abbreviations

BMI	Body Mass Index
CD	Cluster of differentiation
CHD	Coronary heart disease
CK7	Cytokeratin 7
CRD	Carbohydrate recognition domain
DM2	Diabetes Mellitus Type 2
EVT	Extra-villous trophoblast cells
g	Gramm
GDM	Gestational Diabetes Mellitus
h	Hours
IL	Interleukin
INF	Interferon
IRS	Immunoreactivity score
IUGR	Intra uterine growth restriction
MMP	Matrix metalloproteinase
oGTT	Oral glucose tolerance test
PE	Preeclampsia
PP-13	Placental protein 13
SCT	Syncytiotrophoblast
Th	T-helper-cell
TIMP-1	Tissue inhibitor of metalloprotease 1
TNF	Tumor necrosis factor
VEGF-A	Vascular endothelial growth factor A
